# Measuring the Value of Mortality Risk Reductions in Turkey

**DOI:** 10.3390/ijerph110706890

**Published:** 2014-07-04

**Authors:** Cem Tekeşin, Shihomi Ara

**Affiliations:** 1 Department of Economics, Hacettepe University, Ankara 06800, Turkey; E-Mail: cemtekesin@hacettepe.edu.tr; 2 Department of Agricultural, Food and Resource Economics, Michigan State University, East Lansing, MI 48824, USA

**Keywords:** choice experiment, value of statistical life, senior discount, cancer premium, Turkey

## Abstract

The willingness to pay (WTP) for mortality risk reduction from four causes (lung cancer, other type of cancer, respiratory disease, traffic accident) are estimated using random parameter logit model with data from choice experiment for three regions in Turkey. The value of statistical life (VSL) estimated for Afsin-Elbistan, Kutahya-Tavsanli, Ankara and the pooled case are found as 0.56, 0.35, 0.46 and 0.49 million Purchasing Power Parity (PPP) adjusted 2012 US dollars (USD). Different types of risk cause different VSL estimates and we found the lung cancer premium of 213% against traffic accident. The effects of one-year-delayed provision of risk-reduction service are the reduction of WTP by 482 TL ($318 in PPP adjusted USD) per person on average, and the disutility from status-quo (zero risk reduction) against alternative is found to be 891 TL ($589 in PPP adjusted USD) per person on average. Senior discounts of VSL are partially determined by status-quo preference and the amount of discount decreases once the status-quo bias is removed. The peak VSL is found to be for the age group 30–39 and the average VSL for the age group is 0.8 million PPP adjusted USD). Turkey’s compliance to European Union (EU) air quality standard will cause welfare gains of total 373 million PPP adjusted USD for our study areas in terms of reduced number of premature mortality.

## 1. Introduction

The Value of Statistical Life (VSL) has been estimated in various parts of the world and adopted in cost benefit analyses and policy evaluations involving potential direct or indirect changes in mortality risks [1]. Turkey, as a candidate country for European Union membership, is required to adopt sets of EU directives, and as a developing country experiencing rapid industrialization and urbanization, is not an exception of the countries that seek proper and complete country-specific analyses of policy options. Although an Environmental Impact Assessment is mandatory for the projects affecting environment, our review of existing reports revealed that insufficient impact analyses of human health have been conducted in such assessments. It is also often the case that the environmental or health benefit or cost values used in the analyses are borrowed from the case studies conducted in EU countries and are not Turkey-specific values. When a cost benefit analyses (CBA) and an environmental impact assessment regarding a policy or project that affect human mortality is conducted, it is essential to translate the potential increase or decrease in premature mortality into a monetized fashion, since only doing so allows one to conduct a comprehensive CBA and make informed decisions that lead to efficient resource allocations. 

Estimation of VSL allows us to convert a change in mortality risk into a specific monetary value which can be included in CBA. Since no study exists, to the best of our knowledge, estimating the value of statistical life for Turkey, it was impossible to conduct a Turkey-specific complete cost-benefit analysis or health/environmental risk assessment that evaluate the changes in the mortality of the Turkish people. This study attempts to fill in the missing components in CBA for the projects and policies conducted in Turkey. The main objectives of this study were: (1) to estimate VSL for Turkish population using a choice experiment, (2) to measure the impact of risk types on VSL, (3) to observe VSL values for different age groups, and (4) to test the feasibility of conducting a choice experiment in Turkey.

The rest of the article is structured as follows: Section 2 provides an overview of the basic characteristics of the study sites. Section 3 reviews existing related studies. Section 4 describes the survey concept, design, implementation, descriptive statistics and estimation models. Section 5 reports the estimated results for seven models. Section 6 presents an application of the derived VSL in an air pollution policy evaluation setting, followed by the conclusion and discussion in Section 7.

## 2. Description of Study Areas

**Figure 1:**
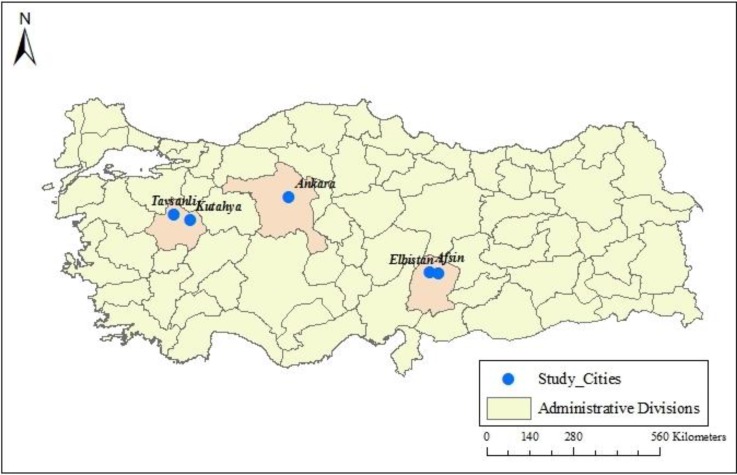
Geographical Location of the Study Areas in Turkey.

We conducted a survey in five cities (Afsin, Elbistan, Kutahya, Tavsanli and Ankara) in three provinces (Ankara (Ankara), Kahramanmaras (Afsin, Elbistan) and Kutahya (Kutahya, Tavsanli)) that are shown in Figure 1. The choice experiment reported in this study is one of the components of a project that is designed to estimate the monetized health benefits of air quality policy, focusing on PM_10_ reduction. We have selected study cities based on the PM_10_ levels up to year 2007, the existence of coal-fired electric power plants, and the socio-economic development ranking.

Based on the 2011 socio-economic development ranking [2], Ankara is the second, Kutahya is the 38th and Kahramanmaras is the 60th out of 81 cities. Ankara (population: 4.9 million, 2011 Census [3] is an example of large and developed cities, Kutahya (population: 0.56 million) is of medium sized cities, and Tavsanli (population: 101,001) in Kutahya, Afsin (population: 84,244) and Elbistan (population: 139,046) in Kahramanmaras are of small sized cities.

While the 1-year average of the EU air quality standard for particulate matter less than 10 micrometer in diameters (PM_10_) is 40 µg/m^3^, in Turkey the level was 150 µg/m^3^ till the end of 2013. Currently, the standard level is targeted to be lowered to 60 µg/m^3^ and it will be lowered to the EU standard by year 2019. The PM_10_ levels especially in Afsin-Elbistan have been high (132, 108 and 114 µg/m^3^ in 2008, 2009 and 2010, respectively), and in winter air pollution has been causing serious health damages in the area due to the lack of natural gas supply of the area and an excess use of low-quality coal for house heating. Winter time (November–February) average PM_10_ levels are 188, 133 and 145 µg/m^3^ in 2008, 2009 and 2010, respectively. Kutahya, on the other hand, has a supply of natural gas since 2005. Prior to 2005, the air pollution was also a serious problem in Kutahya. However, since the introduction of natural gas, air pollution has been decreasing in the region. Other common sources of air pollution in Afsin-Elbistan and Kutahya are the lignite fired electric power plants and lignite open-pit mines within 25 km from each city center (approximately 10 km from Tavsanli to Tuncbilek power plant, 25 km from Kutahya center to Seyitomer power plant, 15 km from Afsin to Afsin-Elbistan power plant and 20 km from Elbistan to Afsin-Elbistan power plant). The result of our study contributes to the estimation of the health benefit from the compliance to the EU air quality standard in terms of reduced premature mortality.

The demographic statistics of the cities’ population are obtained from the Turkish Statistical Institute (TUIK). The male-female ratios of all study areas are about 50.6%, the age group ratio for the ages 20–29, 30–39, 40–49, 50–59, 60–69, and 70–74 are approximately 32%, 25%, 19%, 12%, 9% and 2%, respectively. There are no significant differences in the age structure across study areas. Monthly household disposable income distribution for general Turkish population (household income data are not available for any of our study areas) is reported as 715 TL, 1215 TL, 1726 TL, 2434 TL and 4983 TL as the average values of the 1st through the 5th quintiles [3]. For urban areas, the average values for the same quintiles are 865, 1388, 1921, 2666 and 5498 TL and for rural areas, they are 520, 908, 1274, 1862 and 3517 TL. As for the illiteracy rate, it is 1.7% for males and 8.1% for females among the 15 year-old and older population in Turkey in general. The illiteracy rates for our study areas are [male/female: 2.1%/10.3%] for Kahramanmaras, [0.8%/3.8%] for Kutahya, and [0.7%/4.7%] for Ankara. The higher illiteracy rates for females are common in all study areas.

Approximately 60% of the population in Turkey has elementary school education as their highest degree. Even in Ankara, the second largest city in Turkey, the majority of the population (43% for male, 49% for female) have middle school or less education. The proportion of university graduates is the highest in Ankara (22% for male, 18% for female) and the lowest in Kahramanmaras (14% for male, 8% for female) among our study areas. This high illiteracy rates and relatively low levels of education on average make mail survey and internet survey a non-reliable option in Turkey.

## 3. Literature Review

There is no prior study, to the best of our knowledge, estimating willingness to pay for mortality risk reductions and the value of statistical life in Turkey. We found about ten studies estimating VSLs by using the stated preference method (SP) in developing countries, and those include six studies for China, two studies for Thailand, one for India and one for Mongolia. We will review these studies in the first part of the literature review. There are a few studies deriving VSL by using choice experiment, and we will briefly review these studies in the second part.

### 3.1. A Review of VSL Studies Using SP Method in Developing Countries

Estimated values of statistical life based on specific diseases in developing countries range from $4200 (in 1999 USD) in Anquing, China (Hammitt and Zhou [4]) to $1.32 million (in 2003 USD) in Bangkok, Thailand (Vassanadumrongdee and Matsuoka [5]). Compared to the values used in developed countries (e.g., $7.4 million (in 2006 USD) for USA [6], €1.4 million (in 2000 Euros) for EU countries [7], $1.5–4.5 million (in 2005 USD) with the base value of $3 million for OECD countries and $1.8–5.4 million (in 2005 USD) with the base of $3.6 million for EU-27 [8]), VSL estimates are in general significantly lower in developing countries.

The lowest bound of VSL estimates were found by Hammitt and Zhou [4]. Their study is based on a large (total 3700) sample from three cities, Beijing (a large city), Anqing (a small city) and rural area near Anquing in China. WTP for cold, chronic bronchitis and mortality risk reduction related to air pollution were estimated. For mortality risk reduction, WTPs to reduce mortality risk in next year by 10 or 20 in 10,000 from current risk of 70 in 10,000 were estimated. The median WTP for rural areas ranged between $7830 and $40,500, it was between $4200 and 4780 for Anqing, and it was in the range of $16,300 and $16,900 for Beijing. It is worthwhile to note that the WTP values derived were larger in the rural area than Anqing although the monthly household income was twice as much in Anqing. Vassanadumrongdee and Matsuoka [5] conducted two contingent valuation method (CVM) surveys in Bangkok, Thailand and estimated VSLs based on WTPs for the reduction of mortality risks from air pollution induced lung diseases and traffic accidents. The estimated ranges of VSLs were $0.74–1.32 million (in 2003 USD) for air pollution and $0.87–1.48 million for traffic accidents. 

Other VSL studies on developing countries include Hoffmann *et al.* [9], Bhattacharya *et al.* [10], Wang and Mullahy [11] and Gibson *et al.* [12]. Hoffmann *et al.* [9] conducted a computerized CVM survey in Ulaanbaatar, Mongolia in 2010 and derived the WTP for mortality risk reduction for contemporaneous and latent diseases. The survey was designed to be consistent with seven other studies conducted in the United States, Canada, the United Kingdom, France, Italy, Japan and China [13,14,15,16] and adopted a generic product as the means of reducing the risk. The obtained VSL was $221,000 (in 2009 USD) or $493,000 in PPP adjusted value. Bhattacharya *et al.* [10] conducted CVM to elicit the WTP for reducing the private risk of dying in the context of traffic safety in Delhi, India in 2005. The estimated VSL was $150,000 in PPP adjusted value. Wang and Mullahy [11] estimated the value of saving one statistical life through improving air quality in Chongqing, one of the largest cities in China in 1998. They found that the respondents’ average median WTP to save one statistical life was $34,458. Gibson *et al.* [12] estimated VSL based on landmine clearance context in rural Thailand by using CVM in 2003. Respondents were asked to reveal the amount of income that would make two areas with different risk of death from a landmine accident indifferent. Median VSL was estimated as $295,500. 

Although the relationship between the VSL (WTP for risk reduction) and the respondents’ age was also investigated in the above mentioned studies, the findings are mixed. An inverse-U relationship (VSL increases until its peak, then decreases as an individual gets older) was confirmed in Vassanadumrongdee and Matsuoka [5], while a negative relationship was found in Hammitt and Zhou [4] (a quadratic relationship was not confirmed) and Gibson *et al.* [12], and a positive relationship was found in Wang and Mullahy [11]. In addition, the relationship between the status-quo preference and age was found to be U-shaped in Bhattacharya *et al.* [10].

### 3.2. A Review of VSL Studies Using Choice Experiment

Although the VSL studies reviewed in the previous section use CVM, there are some studies estimating VSL using a choice experiment. We review the VSL-choice experiment studies in this sub-section. Tsuge *et al.* [17] estimated VSL using a choice experiment based on a survey conducted in Tokyo, Japan in 2002. Their attributes included the amount of payment for 10 years, risk reduction for 10 years, risk type (accident, cancer, heart disease and general risk) and the starting date of the risk reduction (now, 5 years or 10 years later). Although they did not find any significant evidence of the differences in people’s WTP for the amount of risk reduction depending on the risk types, people’s WTP for the opportunity of purchasing specific type of risk reduction actually changed according to the risk types. Other findings included negative WTP for risk reduction and strong status quo preference of elderly individuals (over 70).

Itaoka *et al.* [18] conducted a choice experiment to estimate WTP for mortality risk reduction due to different types of electric power generation (fossil fuel *vs.* nuclear power). Alberini *et al.* [19] used choice experiment to estimate VSL in the context of contaminated site cleanup policies in four cities in Italy in 2005. Their design of the profiles included five attributes (lives saved, the size of population affected by the program, number of years until the risk reduction is incurred, number of years the health benefits last and one-time tax payment for the household). Their findings included a higher WTP for males, and no significant differences were found among those who had college degrees. Age was also found to be a statistically insignificant effect on the value of risk reduction.

Carlsson *et al.* [20] compared VSL values for different causes, drowning, fire and traffic accidents by using a choice experiment conducted in Sweden in 2007. Each cause of premature deaths was asked separately and each respondent completed a total of nine choice experiments including three attributes, baseline risk, risk reduction and cost. Notable results included a negative VSL-baseline risk relationship found and an inverse U-shaped VSL-age relationship whose turning points were between 39 and 48 years depending on the accident types.

The study by Cameron and DeShazo [21] utilized a choice experiment with 12 different risk types (prostate cancer, breast cancer, colon cancer, lung cancer, skin cancer, heart disease, heart attack, stroke, respiratory disease, diabetes, Alzheimer's disease and traffic accident) and analyzed the effects of latency, future timing, duration of morbidities and morbidity types on individuals’ WTP on health risk reduction. Among major findings, it is notable to observe the age—WTP for micro risk reduction relationship varied dramatically depending on latency, the duration of morbidity, and premature mortality.

## 4. Choice Experiment

### 4.1. Conceptual Model

The respondent selects an alternative *i* from *J* alternatives. The utility of person *k* from alternative *i* can be written as:

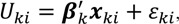
(1)
Where *x_ki_* are observed variables for individual *k* and alternative *i* which include alternative and individual characteristics, and *β_k_* is a vector of coefficients for these variables for individual *k*. In order to accommodate preference heterogeneity among individuals, we assume that the coefficients vary across different individuals with density *f*(*β*). *ε_ki_* is defined as a random component that is an iid extreme value. The probability of an individual *k*’s choosing alternative *i* among other alternatives *j* (*i* ≠ *j*) is expressed as:

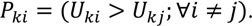
(2)


If we assume that *f*(*β*). is normally distributed (*ϕ*) with mean µ and covariance Σ, the above probability can be rewritten as:

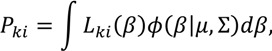
(3)


Where *L_ki_* = 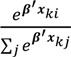.

The parameters are estimated with simulation by maximizing the simulated log likelihood function:

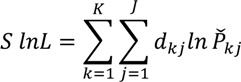
(4)


Where *d_kj_* = 1 if the alternative i is selected and 0 otherwise. 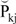 is a simulated probability and is defined as, 
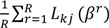
, where R is the number of draws [22]. We use Halton sequences and R = 1000 for the estimation. The marginal WTP for the attribute is derived as the ratio of the estimated coefficient for the specific attribute to the estimated parameter for the cost given the linear utility function.

### 4.2. Survey Design

The survey is composed of four sections. The first section asks basic demographic information, health status and history of minor and major illnesses of the respondent and the close family members (partner, children and parents, separately), observations and opinions of air pollution in the city, and two questions for probability comprehension. The second section includes a CVM question for evaluating the WTP for avoiding chronic bronchitis (the result of CVM is not reported here). The third section is a choice experiment (CE) question with follow-up questions including importance ranking among attributes and risk perceptions on voluntariness, controllability, severity and private knowledge. Monthly net household income, monthly interest payments, annual investment earnings are asked at the end of the survey. The total length of the survey is 14 pages and each questionnaire is completed in about 30 min. 

Prior to the choice experiment, some facts about the mortality from different causes in Turkey are explained. The exact wordings, translated from Turkish to English, are reported in Appendix A. For clearer risk communication, a 10,000 human-shaped-grid (an example is shown in Appendix B) is presented for the explanation of the amount of risk reduction. Interviewers explain the relevant risk reductions (1, 3, 5 or 8/10,000) by using the template prior to the choice experiments. Following the risk descriptions, respondents are presented the CE questions. Two options with five attributes (Risk Type, Affected Person, Effective Date, Risk Reduction, and Price) that propose the possible types of mortality risk reduction from specific causes are explained. They are given the option of “neither”, which is equivalent to selecting the status-quo option. The levels of attributes are presented in Table 1. Four types of risk are selected (1) to reveal the marginal willingness to pay (MWTP) for avoiding respiratory disease in line with health impacts from air-pollution, (2) to examine the cancer premium in our sample, (3) to observe if different MWTP are found for different types of cancers (or equivalently, to see whether the VSL derived from different types of cancer actually changes) and (4) to see if the MWTP is higher for lung cancer which is the major cause of death among various types of cancer (a detailed description of each disease was not provided to the respondents, except for the types of respiratory diseases included in “respiratory disease”. Therefore, the interpretations of “Cancer”, “Traffic Accident” *etc.*, may vary among respondents). Traffic accident is also included by assuming that it is the commonly recognized risk in the population. The types of *Respiratory Disease* include chronic bronchitis, asthma, respiratory infections, and emphysema. 

The second attribute, *Affected Person*, specifies the beneficiary of the product. *Own* indicates that the risk reduction will be experienced by the respondent herself, and *One of the Children* is used to reveal the parental preference on the risk reduction for one of the children aged under 18. For each CE question, either *Own* or *One of the Children* is included. In this paper, we report the results from the *Own* risk. We asked a total of four CE questions to each individual. If the respondent has no child aged under 18, all four questions were asked regarding own risks. If the respondent has at least one child under 18, then we asked two questions for own risk and two questions for one of their children’s risk to minimize the burden of respondents. The ratios of two (with at least one child)/four (without any child) answers for each study area are 349/146, 265/192 and 159/133 for Afsin-Elbistan, Kutahya-Tavsanli and Ankara, respectively. The third attribute, *Effective Date* presents the starting date of risk reduction when the specific risk reduction program is selected.

**Table 1:** Description of Attributes for CE Question.

Attributes	Levels			
Risk Type	Lung Cancer	Other Type of Cancer	Respiratory Disease	Traffic Accident
Affected Person	Own	One of the Children		
Effective Date	Today	1 year later		
Risk Reduction (for 1 year)	1/10,000	3/10,000	5/10,000	8/10,000
Price (for 1 year)	200 TL	400 TL	600 TL	800 TL

### 4.3. Data Collection

Followed by multiple focus-group discussions and pre-tests (September 2011, May 2012) in three cities, the main survey was conducted by trained interviewers during the June–July 2012 period in each city. The total numbers of surveys collected are 501, 531 and 301 in Afsin-Elbistan, Kutahya-Tavsanli and Ankara, respectively (total 1333). After eliminating coding errors and protests, the final numbers of observations included in our analysis are 496, 460 and 292 for Afsin-Elbistan, Kutahya-Tavsanli and Ankara, respectively (total 1248).

Table 2 summarizes the basic demographic characteristics of the respondents from each city. Male over-represents Afsin-Elbistan sample while it is nearly equal in the other two areas. Since Afsin-Elbistan are relatively more conservative cities compared to other cities, male interviewers had some difficulties finding female respondents that agreed to complete the survey (10 interviewers, five male and five female were recruited to conduct both the pre-tests and the main survey in all study locations). When both male and female were present at the time of the interview, the male typically answered the questions. These factors have resulted in the higher male responses in Afsin-Elbistan. The average age of the respondents is about 40 years old, and our sample over-represents the thirties and forties age-groups and under-represents the twenties when compared with the population statistics. The educational background shows large differences between Ankara and the other two locations. While more than 30% of our sample have graduated from universities in Ankara, it is about 10% in Afsin-Elbistan and Kutahya-Tavsanli. Approximately 40% of respondents from Afsin-Elbistan and Kutahya-Tavsanli had at most elementary school education. When we compare the population statistics to the sample regarding education, it becomes clear that our sample is over-educated (higher high school and university graduates ratios) compared to their corresponding population. 

The summary of the counts of major illnesses in each study area is listed in Table 2. The significantly higher occurrence of asthma and chronic bronchitis are observed in Afsin-Elbistan, including the respondents themselves and at least one of their children.

**Table 2:** Descriptive Statistics for Socio-Demographic Characteristics.

City		Afsin-Elbistan		Kutahya-Tavsanli		Ankara	
Variables		Count/Av	Share	Count/Av	Share	Count/Av	Share
Sample Size		496		460		292	
Gender	Male		60%		54%		48%
Age	Average	**40.4**		**42.7**		**42.7**	
	18–19		1%		1%		0%
	20–29		22%		15%		13%
	30–39		29%		28%		30%
	40–49		23%		25%		29%
	50–59		14%		19%		20%
	60–69		9%		8%		7%
	70–75		2%		3%		2%
Numbers of Children	Average	**1.42**		**0.98**		**0.89**	
Education	No Education		5%		4%		2%
	Elementary School		39%		32%		17%
	Middle School		17%		18%		15%
	High School		27%		35%		35%
	University		12%		11%		32%
Income	Average	**1770**		**1825**		**2796**	
	<=650		8%		1%		1%
	651–1000		27%		26%		7%
	1001–2000		32%		45%		26%
	2001–3000		16%		18%		33%
	3001–4000		4%		7%		17%
	4001–5000		3%		2%		8%
	>=5001		4%		2%		9%
Chronic Diseases (Own)	High Blood Pressure	82	17%	66	14%	40	14%
	High Cholesterol	53	11%	42	9%	37	13%
	Diabetes	38	8%	45	10%	19	6%
	Asthma	77	16%	35	8%	18	6%
	Chronic Bronchitis	49	10%	22	5%	15	5%
	Heart Disease	56	11%	51	11%	25	9%
	Cancer	6	1%	2	0.4%	1	0%
Chronic Diseases (Partner)	High Blood Pressure	50		56		35	
	High Cholesterol	29		39		27	
	Diabetes	38		39		23	
	Asthma	42		22		15	
	Chronic Bronchitis	29		10		6	
	Heart Disease	32		48		21	
	Cancer	4		3		1	
Chronic Diseases (At least one of the children per family)	Asthma	38		16		18	
	Chronic Bronchitis	63		17		8	
	Heart Disease	6		10		5	
	Cancer	1		1		0	
To understand hypothetical. questions	Very Difficult, Difficult		18%		14%		11%
	Very Easy, Easy		81%		85%		88%
Confidence of answers for hypothetical Qs	Very Confident, Confident		90%		92%		95%
	Not confident, Don’t know		10%		6%		5%

While asthma is experienced by 8% and 6% of the respondents in Kutahya-Tavsanli and Ankara, respectively, it is 16% in Afsin-Elbistan. Sixty-three households (17%) of Afsin-Elbistan have at least one child that experienced asthma out of 365 households that have at least one child under 18. While the rate of experiencing chronic bronchitis is 5% in Kutahya-Tavsanli and Ankara, it is 10% in Afsin-Elbistan. 

It is critical to know the level of understanding and confidence in respondents’ answers especially for the case of surveys which require good comprehension of small probability and hypothetical questions conducted in developing countries. More than 80% of the respondents stated that understanding hypothetical questions were “easy” or “very easy” and 90% or more answered that they are confident about their answers on the amount of payment they had agreed to pay for the hypothetical questions. Furthermore, close to 90% of the respondents answered the question which test the comprehension of small probability correctly (probability comprehension questions: (1). Under which situation do you have the higher risk of getting sick? (A. 5 out of 10,000 person getting sick, B. 10 out of 10,000 person getting sick), (2). Which situation do you prefer experiencing, A or B?). Therefore, we can conclude that it is quite feasible to conduct stated preference survey which is based on hypothetical questions with small changes in probabilities even in small cities in Turkey, as long as it is conducted via face-to-face interviews.

As for the monthly household income, Ankara households earn on average 2796 TL (1570 USD, using 1 USD = 1.80 TL) monthly, and it is more than 50% higher compared to other study cities. When we rearrange the income data from our sample to the form of TUIK’s distribution of annual household disposable incomes by quintiles ordered by household disposable income, the income distribution of our sample are derived as (622, 711, 1147 TL) for the averages of the 1st quintiles for Afsin-Elbistan, Kutahya-Tavsanli and Ankara, respectively, and similarly, they are (887, 1120, 1975) for the 2nd quintiles, (1267, 1607, 2515) for the third, (1901, 2007, 3308) for the fourth and (3648, 3440 and 5123) for the fifth quintiles. Afsin-Elbistan values are approximately 30% lower than the national average and are very similar to the averages for the rural areas, Tavsanli-Kutahya values are quite close to the national average from the 1st to the 3rd quintiles, but 20% higher for the 4th and 45% higher for the 5th quintiles, and for the Ankara sample, our sample is approximately 45% higher than the national average and 25% higher than the averages for urban areas in each quintile. 

Four questions on subjective perception of risks are asked after the set of choice experiment questions. The questions are on perceived mortality risk, individual responsibility, private knowledge, and the role of public policy (Table 3). Respiratory disease is perceived to have the lowest mortality risk while it is the highest for lung cancer. Personal responsibility is considered to be the highest for traffic accident for Afsin-Elbistan and Ankara and it is lung cancer in Kutahya-Tavsanli. More than 80 percent of the respondents in Afsin-Elbistan and Kutahya-Tavsanli believe that it is the government’s responsibility to reduce the risks of lung cancer, respiratory disease and traffic accident. It also became clear that the perceived risk from “Other type of Cancer” might be ambiguous among respondents as we observe the lowest proportion of agreement for private knowledge for all study areas. 

**Table 3:** Subjective Perception of Risks (% of “Agree” and “Strongly Agree” Responses).

City\Risk Type	Lung Cancer	Cancer	Respiratory Disease	Traffic Accident
	“Mortality rate is high for this risk”			
Afsin-Elbistan	96%	90%	83%	90%
Kutahya-Tavsanli	96%	94%	79%	95%
Ankara	99%	94%	77%	97%
	“I am responsible for the cause of this risk”			
Afsin-Elbistan	64%	54%	64%	72%
Kutahya-Tavsanli	77%	65%	76%	73%
Ankara	74%	59%	70%	75%
	“I know the cause of this risk very well”			
Afsin-Elbistan	72%	61%	74%	79%
Kutahya-Tavsanli	75%	63%	82%	85%
Ankara	77%	66%	82%	86%
	“Public policy can reduce this risk”			
Afsin-Elbistan	86%	73%	86%	84%
Kutahya-Tavsanli	81%	73%	83%	82%
Ankara	79%	63%	78%	87%

### 4.4. Estimation Models

In this article, we report the estimated results for the following eight specifications. The definitions of all variables are listed in Table 4. Model 1 is our base model with only the attribute variables and the status-quo constant. The risk type coefficients are used to derive the WTP for risk reduction by a specific cause regardless of the amount of risk reduction:

*V* = *β*_0_*SQ + β*_1_*PRICE + β*_2_*RISK + β*_3_*DATE + β*_4_*LUNG + β*_5_*CANCER + β*_6_*TRAFFIC* (Model 1)

Model 2 is designed to reveal the changes in the valuation of 1 in 10,000 risk reduction depending on the different risk types. Statistically significant estimates for β_3_, β_4_ and β_5_ indicate the differences in willingness to pay for 1 in 10,000 risk reduction caused by different types of risks. The baseline risk type is respiratory disease and is expressed as β_2_:

*V = β*_0_*SQ + β*_1_*PRICE + (β*_2_* + β*_3_*LUNG + β*_4_*CANCER + β*_5_*TRAFFIC)RISK + β*_6_*DATE + β*_7_*LUNG + β*_8_*CANCER + β*_9_*TRAFFIC* (Model 2)

Model 3 and Model 4 include the first set of individual characteristics as the interactions with the status-quo or risk to observe the influence of demographic parameters (*GENDER*, *AGE*, *AGE^2^ and HHINC*) on the status-quo or risk evaluation. Model 5 and Model 6 repeat the idea of Model 3 and Model 4, but with the second set of individual specific variables (*UNIV*, *OVER65*, *ASTCB*, *CVASC*, *HTCOAL*, *GOODHLTH*). We separate them into two models to avoid high correlation among demographic variables. Model 7 and Model 8 are intended to analyze the age-risk relationship and the age-status-quo preference. Since the result of Model 3 suggests an inverse U relationship between age and the valuation of risk reduction, nine different age groups (*AGE1824*, *…* , *AGE6575*; *AGE3034*) is dropped for Afsin-Elbistan and Kutahya-Tavsanli, and *AGE3439* is omitted for Ankara) are included by setting the peak age group of inverse-U as the base variable.

**Table 4:** Variable Descriptions.

Variable	Description
*Attribute variables*	
PRICE	200, 400, 600 or 800 TL
RISK	1,3,5 or 8/10,000 mortality risk reduction over 1 year
DATE	0 if risk reduction starts today, 1 if it starts one year from now
LUNG	1 if lung cancer, 0 otherwise
CANCER	1 if cancer except for lung cancer, 0 otherwise
TRAFFIC	1 if traffic accident, 0 otherwise
ASC_SQ	Alternative specific constant for status quo
*Demographic and attitudinal variables, interacted with ASC_SQ or RISK.*	
HHINC	Monthly household income /1,000
GENDER	1 if the respondent is a female, 0 otherwise
AGE	Age of the respondent
UNIV	1 if having university or higher degree, 0 otherwise
OVER65	1 if the respondent is 65 and over, 0 otherwise
ASTCB	1 if the respondent has experienced (experiencing) Asthma or Chronic Bronchitis in last three years, 0 otherwise
CVASC	1 if the respondent has experienced (experiencing) Cardio-Vascular disease in last three years, 0 otherwise
HTCOAL	1 if coal is used as the main source of household heating, 0 otherwise
GDHLTH	1 if the respondent consider she is in good health , 0 otherwise
AGE1824	1 if the respondent is between 18 and 24 years old, 0 otherwise. (Other age dummy variables are defined similarly.)

## 5. Results

### 5.1. General Results for Adults

All models are estimated with the NLOGIT 4.0 software by the random parameter logit model using 1,000 repetitions for simulated probabilities with Halton draws. We specify the PRICE, ASC_SQ and all variables with cross-terms as fixed parameters. The RISK, DATE, LUNG, CANCER and TRAFFIC coefficients are allowed to vary and are assumed to be normally distributed. The estimated results for Model 1 are presented in Table 5 for each city and the pooled data.

**Table 5:** Estimated Results for Model 1.

	Model 1							
Mean Parameters	Afsin-Elbistan		Kutahya-Tavsanli		Ankara		Pooled	
RISK	0.518	***	0.577	***	0.403	***	0.556	***
	(0.152)		(0.222)		(0.124)		(0.103)	
DATE	−2.851	***	−5.553	***	−2.723	***	−3.669	***
	(0.586)		(1.198)		(0.571)		(0.453)	
LUNG	2.561	***	4.439	***	3.850	***	3.315	***
	(0.560)		(1.164)		(0.859)		(0.443)	
CANCER	1.236	***	1.528	**	2.522	***	1.807	***
	(0.464)		(0.771)		(0.652)		(0.366)	
TRAFFIC	−4.399	***	−10.748	***	−2.465	***	−5.653	***
	(1.121)		(2.601)		(0.837)		(0.893)	
*Fixed Parameters*								
ASC_SQ	−5.691	***	−9.308	***	−5.158	***	−6.590	***
	(0.991)		(1.939)		(1.046)		(0.713)	
PRICE	−0.006	***	−0.011	***	−0.006	***	−0.008	***
	(0.001)		(0.002)		(0.001)		(0.001)	
*Standard Deviation Parameters*								
RISK	1.316	***	3.096	***	1.056	***	1.735	***
	(0.249)		(0.661)		(0.212)		(0.196)	
DATE	2.484	***	3.563	***	2.085	***	2.604	***
	(0.618)		(1.078)		(0.537)		(0.408)	
LUNG	2.415	***	3.404	***	3.093	***	2.886	***
	(0.844)		(1.143)		(0.985)		(0.623)	
CANCER	3.800	***	9.195	***	3.777	***	5.459	***
	(1.068)		(2.468)		(0.981)		(0.832)	
TRAFFIC	6.940	***	13.767	***	5.280	***	8.640	***
	(1.666)		(3.306)		(1.279)		(1.256)	
Log likelihood	−995		−955		−620		−2609	
McFaaden Pseudo R2	0.29		0.33		0.34		0.31	
Number of Observations	1283		1305		850		3438	

***, **, and * stand for the statistical significance at 1%, 5% and 10% levels, respectively. The values inside parentheses are standard errors.

In Model 1, all variables are statistically significant at least at the five percent level. All statistically significant estimates are with expected signs, *i.e.*, negative for PRICE and DATE, positive for RISK, indicating the preferences for lower price, earlier and higher risk reductions. The statistically significant estimates for standard deviation parameters indicate that these parameters actually vary in the population. The variables for the causes of premature mortalities, LUNG (lung cancer) and CANCER (other cancer) are positive, while TRAFFIC is negative and significant. The base line risk type is the respiratory disease. This result suggests that the WTPs for reducing risk caused by the lung cancer or other type of cancer are higher (regardless of the amount of risk reduction) than the respiratory disease case and is lower than the traffic accident. Furthermore, it is revealed that different amount of premiums are given to different types of cancers. The ranking of the premiums (1. lung cancer, 2. other type of cancer, 3. respiratory diseases, 4. traffic accident) coincides in all three cities. 

When we compare the results across the cities, the different characteristics observed for Kutahya-Tavsanli become clear. Risk reduction is evaluated the most and postponed risk reduction for one year (DATE) is devaluated the most in Kutahya-Tavsanli. However, the respondents in Kutahya-Tavsanli are more sensitive to the price of the risk reduction package. As a result, when we calculate the willingness to pay for 1 in 10,000 risk reduction and the value of statistical life, the results for Kutahya-Tavsanli are recorded as the lowest. Here, we observe the discrepancy between the risk perception and willingness (capability) for payment. This tendency could also be observed from the difference in the out-of-pocket monthly medical costs for chronic illnesses in these cities. The average monthly out-of-pocket medical cost is 72 TL (maximum 7500 TL) in Afsin-Elbistan while it is 40 TL (maximum 3000 TL) in Kutahya-Tavsanli in our sample. Kutahya-Tavsanli respondents care about the differences of the causes of the mortality risk reductions the most as well.

The alternative specific constants for the status quo (ASC_SQ, coded as 1 for status-quo and 0 otherwise) are negative and significant for all models. This implies the disutility against the status-quo or the no-risk reduction situation. Such disutility is the highest in Kutahya-Tavsanli and the lowest in Ankara. The PRICE variable is also negative and statistically significant as expected, meaning an increase in the price of the mortality risk reduction decreases the probability of an alternative profile to be chosen as oppose to the status quo. 

The point estimates of marginal WTP and the estimated VSL are reported in Table 6, based on the estimated results of Model 1 and using the TL-USD exchange rate as of July 2nd, 2012 (1 USD = 1.80 TL) and the purchasing power parity (PPP) of TL × 1.189 by using the 2011 PPP values reported in the OECD.StatExtracts database [23]. The standard errors are estimated by using 10,000 repetitions of the Krinsky and Robb method [24]. The values of 1 in 10,000 reduction of mortality risk are calculated as 85 TL ($47), 53 TL ($29), and 69 TL ($38) for Afsin-Elbistan, Kutahya-Tavsanli, and Ankara, respectively. Although the risk reduction is evaluated the highest in Kutahya-Tavsanli, due to the price sensitivity, the calculated MWTP for 1 in 10,000 risk reduction becomes the lowest among three cities. The risk reduction that will start after one year decreases the WTP by 471 TL ($262), 508 TL ($282), and 466 TL ($259) for Afsin-Elbistan, Kutahya-Tavsanli and Ankara, respectively, compared to the immediate risk reduction. This result suggests that the discount rate for the risk reduction is the highest in Kutahya-Tavsanli and the lowest in Ankara. Although we cannot compare the magnitudes of cancer premiums directly across different cities since the LUNG, CANCER and TRAFFIC variables are dummy variables, we confirmed that people evaluate distinctive types of risk reduction differently regardless of the reduction amount. This is further investigated with Model 2 in the following section. Compared to the alternatives, the status quo option (no reduction in premature mortality risk) is associated with the lower level of utility, and the difference is found to be 940 TL ($522), 851 TL ($473), and 883 TL ($491) in Afsin-Elbistan, Kutahya-Tavsanli and Ankara, respectively. As in the case for RISK estimate, although respondents in Kutahya-Tavsanli seek for the risk reduction alternatives the most, its WTP is estimated the lowest among others due to their price sensitivity. Although transfers of WTP estimates to other parts of Turkey have to be done through well-examined benefits transfers, if we focus on the relationship between the national and sample income distributions, the WTP estimates for Afsin-Elbistan and Kutahya-Tavsanli could be good representatives for the rural areas and the national average, respectively. The WTP for Ankara might be overestimated for urban population. However, as the WTP values for Afsin-Elbistan are higher than the ones in Kutahya-Tavsanli where the income level is higher, it is difficult to generalize our estimates to other areas without taking into account other major factors such as the baseline health and environmental risks.

**Table 6:** Point Estimates for Marginal WTP and Estimated VSL based on Model 1 (TL).

Variable	Afsin-Elbistan		Kutahya-Tavsanli		Ankara		Pooled	
	**WTP**	**S.E.**	**WTP**	**S.E.**	**WTP**	**S.E.**	**WTP**	**S.E.**
RISK	85	21	53	19	69	21	74	12
DATE	−471	70	−508	65	−466	−87	−489	38
LUNG	423	94	406	85	659	188	441	51
CANCER	*204*	89	*140*	82	432	126	241	48
TRAFFIC	−726	173	−983	196	−422	147	−753	103
ASC_SQ	−940	74	−851	44	−883	81	−877	31
VSL (TL)	854,420		527,878		689,104		740,838	
VSL(PPP,$)	564,392		348,692		455,191		489,364	

Standard errors simulated by using 1000 draws of Krinsky and Robb method. The exchange rate ($1 = 1.80 TL) is taken from the Central Bank of Turkey on 2nd July, 2012. PPP value of 2011 (1 TL = 1.189 PPP, TL) is adopted from the OECD.StatExtracts database. Bold estimates are statistically significant at 1% level, Bold-Italic and Italic estimates are statistically significant at 5% and 10%, respectively.

As for the derived VSL, the highest VSL is for the Afsin-Elbistan sample while the lowest is for Kutahya-Tavsanli. As we have discussed earlier, there are two factors affecting the magnitudes of VSL. One is the level of premature mortality risk perception and the other is willingness and capacity to pay for risk reduction. If we use the result from pooled data as the average VSL in these three cities in Turkey, 740,838 TL (489,364 PPP adjusted USD), about half a million dollars is the VSL value we have estimated for Turkey. 

Although our estimated VSL is higher than the VSL estimated in developing countries such as in China ($4000–$17,000 in 1999 USD [4], $34,458 in 1998 USD [11]) and India ($150,000, PPP adjusted 2005 USD, [10]), it is significantly lower than the estimates for developed countries such as $7.4 million (in 2006 USD), the recommended value by the U.S. Environmental Protection Agency for USA [6] and $ 2.9 million in 2002 USD for Japan estimated by Tsuge *et al.* [17]. Our estimate is slightly more than one-fourth of the value adopted by the European Environment Agency (EEA), that is 1.4 million Euro in 2000 Euros (approximately $1.34 million in 2000 USD or $1.72 million in 2012 USD) [7]. It is a half of the middle value of VSL estimates using income-elasticity adjustment ($0.6 million (in 1989 USD) = $0.98 million (in 2012 USD)) and very close to the middle value of VSL estimates using wage-rate adjustment ($0.29 million (in 1989 USD) = $0.47 million (in 2012 USD)) for Central and Eastern Europe derived by Krupnick *et al.* [25] based on benefits transfer using US, Canada and Western European studies. Other studies that estimated a similar VSL as our study are the one for Mongolia ($0.49 million in PPP adjusted 2009 USD, [9]) and the wage-based estimates for Taiwan, $0.4 and $0.6 million in the late 90s [26,27].

Model 2 is intended to reveal the “Cancer Risk Premium” (Table 7). Here, we define the cancer risk premium as the summation of two factors, (i) WTP for the opportunity of reducing the cancer risk (regardless of the degree of risk reduction) and (ii) WTP for reducing the cancer risk per one unit (1/10,000 in our case) of mortality risk reduction [15]. Therefore, the total cancer risk premium that is defined as the difference from the base risk case (respiratory disease) can be calculated as follows:

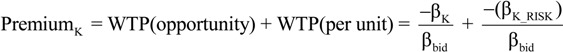
(5)
where *k* corresponds to risk types, LUNG or CANCER, and *Β_K_RISK_* are the coefficients of risk type-risk cross terms (RESP is the base risk type). The first term does not rely on the amount of risk reduction while the second term is the WTP per unit reduction of the mortality risk. For example, the premium for the general cancer risk for Afsin-Elbistan is calculated as 
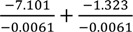
. The difference between the mortality risk reduction from respiratory illness and from traffic accident is also calculated by setting −β_k_ as −β_Traffic_ and β_k_RISK_ as β_Traffic_RISK_. The premium for the larger risk reduction should be calculated as the first term plus the second term multiplied by the size of the risk reduction. The calculated premiums by taking RESP as the base risk type is reported in Table 8. 

The premiums are positive for both lung cancer and the other type of cancer. The negative calculated “Premium” for traffic accident indicates the respondents’ stronger preference for reducing the mortality risk from respiratory illnesses compared to the traffic accident. Distribution of risk-risk trade-offs between the risk of contracting chronic bronchitis (CB) and auto fatality reported in Viscusi *et al.* [28] shows that for the median respondent, the auto death risk was three times more highly valued than the CB risk while the top 20% of the distribution of trade-offs revealed that CB was more highly (up to four times) valued than traffic fatality. Since our study considers mortalities from both risks, we cannot compare the results directly. However, it seems that our respondents value the mortality risks between respiratory illnesses (including CB) and traffic accidents quite differently from their study.

Since we did not provide detailed description of each risk type, respondents’ answers were based on their perceived mortality risks from each risk type. One of the follow-up questions (“The mortality risk of the following risk type is high, do you agree?”) reveals individuals’ perceptions on mortality risks (Table 3). The perceived mortality risk is the highest for Lung Cancer, followed by Traffic Accident and Other Type of Cancer. The calculated differences in the WTP values and the perceived mortality risk for each risk type reveals that the WTP is not solely determined by the level of perceived mortality risk, but also with other factors such as pain, fear, and the duration of suffering. The result shows that our respondents gave a higher weight for non-mortality aspects of respiratory illnesses. The calculated cancer premium for lung cancer is 213% on average, and it is 597% for other types of cancer.

Although there is no study estimating the cancer premium in developing countries, to the best of our knowledge, if we compare our results from the cancer premium estimates for developed countries that range from no premium to 200% premium, it seems quite high. In UK, the cancer premium of 100% is recommended [29] and in the US, it is 50% for their policy assessments [30]. Magat *et al.* [31] found a 58% premium for curable lymph cancer against automobile accident deaths at the mean values, Viscusi *et al.* [32] estimated the cancer premium for fatal bladder cancer from drinking arsenic contaminated water and found a 21% premium compared to the US estimates for acute accidents using a large US sample, and Van Houtven *et al.* [33] found a 200% premium for cancer ($19 million) against immediate fatal accidents ($6 million) when the latency period was 5 years in the US. Hammitt and Liu [34] found a 30% premium for lung or liver cancer compared to the non-cancer lung or liver diseases in Taiwan, while Hammitt and Haninger [35] found no premium in the US. 

The extremely high premium for the general (non-lung) cancer risk is highly likely due to the generality of the “cancer” definition. According to the subjective risk perceptions reported in Table 3, “Other Type of Cancer” earned the lowest percentages of agreement for all four questions, the lowest perceived mortality risk, the lowest personal responsibility, the lowest private knowledge and the lowest responsibility of public policy for all three study areas. This is an indication of the variations in the respondents-defined risk type and it is also possible that some respondents consider multiple types of cancer risks at the same time. In addition, people tend to over-estimate the risk when they are not familiar with it. This result brings us to the conclusion that “cancer risk premiums” have to be determined based on a specific type of cancer, not as general cancer. Therefore, we conclude that 213% of lung cancer premium is a more reliable estimate than the general cancer premium in our study.

**Table 7:** Estimated Result and Risk Type Premium for Model 2.

	Model 2							
Mean Parameters	Afsin-Elbistan		Kutahya-Tavsanli		Ankara		Pooled	
RISK	0.999	***	1.156	***	1.700	***	1.330	***
	(0.322)		(0.337)		(0.609)		(0.288)	
DATE	−3.732	***	−6.913	***	−6.427	***	−5.252	***
	(0.920)		(1.061)		(2.011)		(0.841)	
LUNG	−2.800		−5.673		−3.736		−4.222	**
	(2.032)		(4.423)		(4.279)		(1.683)	
CANCER	7.101	***	7.680	***	12.833	***	8.486	***
	(2.087)		(2.594)		(4.567)		(1.720)	
TRAFFIC	−6.027	***	−9.383	***	0.126		−5.655	***
	(1.998)		(2.655)		(1.976)		(1.368)	
*Fixed Parameters*								
ASC_SQ	−5.562	***	−7.355	***	−6.441	***	−6.210	***
	(1.179)		(2.047)		(2.103)		(0.889)	
PRICE	−0.006	***	−0.009	***	−0.008	***	−0.008	***
	(0.002)		(0.003)		(0.003)		(0.001)	
LUNG*RISK	1.906	***	2.898	**	3.339	**	2.569	***
	(0.714)		(1.345)		(1.648)		(0.610)	
CANCER*RISK	−1.323	***	−1.470	***	−1.797	**	−1.455	***
	(0.413)		(0.479)		(0.747)		(0.334)	
TRAF*RISK	0.090		−0.686	**	−0.806	*	−0.295	*
	(0.205)		(0.298)		(0.425)		(0.167)	
Log likelihood	−979		−940		−605		−2568	
McFaaden Pseudo	0.31		0.34		0.35		0.32	
Number of Obs.	1283		1305		850		3438	

***, **, and * stand for the statistical significance at 1%, 5% and 10% levels, respectively. The values inside parentheses are standard errors. The estimated results of standard deviation parameters are omitted from the table due to the space constraint. All the estimates are statistically significant at one percent level. Available from the corresponding author by request.

**Table 8:** Differences in WTP for Different Risk Types (TL) *.

Risk Type	Afsin-Elbistan	Kutahya-Tavsanli	Ankara	Average
LUNG	314	320	399	344
CANCER	952	685	1317	985
TRAFFIC	−993	−1110	−96	−733

*Calculated based on Model 2 coefficients that are statistically significant at least at 10% level. The base risk type is RESP.

### 5.2. Results for Models with Individual Characteristics

Models 3 and 4 include basic individual characteristics, GENDER, AGE, AGE2 (age squared) and HHINC (monthly household income divided by 1000) as the cross-terms with the status-quo variable (Model 3) or the RISK variable (Model 4). As shown in Table 9, GENDER does not influence the preference over the status-quo or risk valuation. As for age and status-quo preference, there is no significant relationship observed in Afsin-Elbistan and Ankara; but in Kutahya-Tavsanli, we confirmed a weak evidence of a U-shaped relationship, as an individual's age increases the preference for status-quo decreases, but after age 41, it becomes less as she/he gets older. This finding coincides with the one in Bhattacharya *et al.* [10]. In Afsin-Elbistan and Kutahya-Tavsanli, the higher the respondents’ household income is, the less likely the status-quo option is preferred. We did not confirm the same relationship for Ankara. As for the age and household income influences on the risk evaluation, we observed no impact in Afsin-Elbistan and Ankara. The result for Kutahya-Tavsanli reveals that the respondents with higher income tend to evaluate the risk more, as monthly income increases by 1000 TL, WTP for risk reduction, ceteris paribus, will increase by 63 TL. We also confirmed an inverse U-shaped relationship between age and risk, hitting a peak around the age of 38 in Kutahya-Tavsanli. The studies that confirmed an inverse-U relationship include Vassanadumrongdee and Matsuoka [5], Carlsson *et al.* [20] with peaks at 39 and 48 varying by the type of accidents, Aldy and Viscusi [36] with a peak at 46.

The second set of individual characteristics models are estimated as Models 5 (with status-quo cross terms) and 6 (with risk cross terms). The results are listed in Table 10. Since household income is highly correlated with UNIV, separate models are estimated. According to the Model 5 result, university graduates prefer non-status quo options significantly more than non-graduates in Afsin-Elbistan and Kutahya-Tavsanli. We did not observe the same result in Ankara. Certain proportion of university graduates found in our sample in Afsin-Elbistan and Kutahya-Tavsanli have moved from larger cities and have been working as teachers or as assigned government employees. Therefore, in addition to the “education-effect”, such distinct characteristics of university graduates in local cities might have caused the clear differences in the preference for the status quo. In Afsin-Elbistan and Kutahya-Tavsanli, those who are using coal as the main household heating source prefer selecting the status-quo option more than others. In Kutahya-Tavsanli and Ankara, those who have been suffering from asthma or chronic bronchitis prefer the risk reduction options. Those who are over 65 and consider themselves as having good health are more likely to select the status-quo option in Kutahya-Tavsanli. Model 6 considers the risk-individual characteristics interactions. University graduates are willing to pay 101 TL and 111 TL more for the risk reduction and those who use coal as their main heating source evaluate the risk less (equivalent to 60 TL and 67 TL less) than others in Afsin-Elbistan and Kutahya-Tavsanli, respectively. People over 65 are willing to pay less for the risk in Kutahya-Tavsanli, but not so in other two cities. We will investigate this age-risk valuation relationship more in Model 7. The good health condition tends to make people value risk reduction less in Afsin-Elbistan (60 TL less) and Ankara (53 TL less). We did not find any evidence of the influence of existing illnesses on the individual preference over risk reduction from the results based on each city.

**Table 9:** Estimated Results for Model 3 (ASC_SQ Cross Terms) and 4 (RISK Cross Terms).

	Model 3						Model 4					
Mean Parameters	Afsin-Elbistan		Kutahya-Tavsanli		Ankara		Afsin-Elbistan		Kutahya-Tavsanli		Ankara	
RISK	0.455	***	0.467	**	0.356	***	0.325		−2.837		−0.367	
	(0.125)		(0.209)		(0.118)		(0.746)		(1.740)		(0.983)	
DATE	−2.686	***	−5.412	***	−2.328	***	−2.891	***	−5.251	***	−2.945	***
	(0.521)		(1.348)		(0.493)		(0.575)		(1.445)		(0.672)	
LUNG	2.467	***	4.540	***	3.388	***	2.658	***	4.329	***	4.395	***
	(0.526)		(1.325)		(0.841)		(0.581)		(1.524)		(1.137)	
CANCER	1.084	***	1.852	*	2.116	***	1.255	***	1.816	*	2.747	***
	(0.419)		(0.978)		(0.566)		(0.463)		(0.994)		(0.773)	
TRAFFIC	−4.017	***	−10.819	***	−2.033	***	−4.509	***	−10.320	***	−2.539	***
	(0.989)		(3.140)		(0.696)		(1.143)		(3.322)		(0.875)	
*Fixed Parameters*												
ASC_SQ	−5.118	**	0.998		−1.738		−5.947	***	−8.854	***	−5.267	***
	(2.300)		(4.064)		(3.217)		(1.047)		(2.097)		(1.111)	
PRICE	−0.006	***	−0.011	***	−0.005	***	−0.006	***	−0.010	***	−0.006	***
	(0.001)		(0.003)		(0.001)		(0.001)		(0.003)		(0.001)	
Cross with withTerms	ASC_SQ						RISK					
GENDER *	0.090		−0.669		0.433		−0.047		−0.288		−0.034	
	(0.462)		(0.744)		(0.421)		(0.161)		(0.301)		(0.137)	
AGE *	0.046		−0.330	*	−0.174		0.009		0.151	*	0.040	
	(0.098)		(0.188)		(0.146)		(0.035)		(0.082)		(0.047)	
AGE2 *	0.0003		0.004	*	0.003	*	−0.000		−0.002	**	−0.0006	
	(0.001)		(0.002)		(0.002)		(0.000)		(0.001)		(0.0005)	
HHINC *	−1.181	***	−2.589	***	−0.330		0.101		0.646	***	0.086	
	(0.322)		(0.804)		(0.213)		(0.065)		(0.211)		(0.064)	
LogL	−977		−933		−610		−986		−939		−614	
PseudoR^2^	0.30		0.35		0.35		0.30		0.34		0.34	
N. Obs.	1275		1301		850		1275		1301		850	

***, **, and * stand for the statistical significance at 1%, 5% and 10% levels, respectively. The values inside parentheses are standard errors. The estimated results of standard deviation parameters are omitted from the table due to the space constraint. All the estimates are statistically significant at one percent level. Available from the corresponding author by request.

**Table 10:** Estimated Results for Model 5 (ASC_SQ Cross Terms) and 6 (RISK Cross Terms).

	Model 5												Model 6										
Mean Parameters	Afsin-Elbistan				Kutahya-Tavsanli				Ankara				Afsin-Elbistan				Kutahya-Tavsanli				Ankara		
RISK	0.480		***		0.543		*		0.437		***		0.841		***		1.045		***		0.503		**
	(0.130)				(0.279)				(0.153)				(0.268)				(0.396)				(0.199)		
DATE	−2.751		***		−5.563		***		−2.775		***		−3.023		***		−5.938		***		−2.851		***
	(0.534)				(1.262)				(0.683)				(0.628)				(1.219)				(0.683)		
LUNG	2.557		***		4.741		***		4.015		***		2.801		***		4.399		***		4.257		***
	(0.556)				(1.400)				(1.034)				(0.657)				(1.020)				(1.143)		
CANCER	1.108		***		1.543		*		2.497		***		1.287		***		1.657		*		2.604		***
	(0.427)				(0.880)				(0.792)				(0.494)				(0.855)				(0.726)		
TRAFFIC	−4.227		***		−10.691		***		−2.187		***		−4.586		***		−11.389		***		−2.149		***
	(1.048)				(2.821)				(0.792)				(1.198)				(2.574)				(0.764)		
*Fixed Parameters*																							
ASC_SQ	−6.864		***		−10.295		***		−4.840		***		−6.039		***		−9.085		***		−5.134		***
	(1.182)				(2.357)				(1.251)				(1.084)				(1.847)				(1.138)		
PRICE	−0.006		***		−0.011		***		−0.006		***		−0.007		***		−0.011		***		−0.006		***	
	(0.001)				(0.003)				(0.002)				(0.001)				(0.002)				(0.002)			
Cross with	ASC_SQ												RISK											
UNIV	−2.688		**		−3.728		**		−0.765				0.669		**		1.198		**		0.327			
	(1.302)				(1.784)				(0.677)				(0.291)				(0.597)				(0.206)			
OVER65	0.459				8.532		***		1.445				−0.394				−2.680		***		−0.099			
	(0.998)				(2.645)				(1.058)				(0.437)				(0.744)				(0.383)			
ASTCB	0.321				−3.192		**		−5.706		**		0.168				0.539				0.384			
	(0.591)				(1.472)				(2.575)				(0.217)				(0.464)				(0.328)			
CVASC	−0.185				0.524				−0.009				0.344				0.341				0.018			
	(0.701)				(1.177)				(0.993)				(0.285)				(0.452)				(0.305)			
HTCOAL	1.815		***		1.513		*		0.041				−0.395		**		−0.718		**		−0.392			
	(0.612)				(0.823)				(1.590)				(0.198)				(0.354)				(0.390)			
GDHLTH	−0.218				1.498		*		−0.005				−0.395		**		−0.061				−0.314		*	
	(0.497)				(0.843)				(0.558)				(0.193)				(0.351)				(0.191)			
LogL	−983				−925				−612				−983				−940				−615			
PseudoR^2^	0.30				0.35				0.34				0.30				0.34				0.34			
N. Obs.	1283				1301				850				1283				1301				850			

***, **, and * stand for the statistical significance at 1%, 5% and 10% levels, respectively. The values inside parentheses are standard errors. The estimated results of standard deviation parameters are omitted from the table due to the space constraint. All the estimates are statistically significant at one percent level. Available from the corresponding author by request.

### 5.3. Age, Status-Quo Preference and Risk Valuation

Given the insignificant age effects on the valuation of risk reduction confirmed in Model 4 and 6 for Afsin-Elbistan and Ankara, Model 7 and Model 8 with multiple age group dummy variables are tested to further examine the underlying age-status-quo preference and age-risk valuation relationship (Table 11). The findings in the existing studies regarding age-risk valuation (VSL) are mixed [13,16,37,38]. By observing the estimates from multiple trials based on Model 7, we identified the “peak” (all the other estimates have negative signs) of the coefficients among other age groups and dropped the identified age group as the baseline of the age dummy variables. The estimated results of Model 7 indicate that the older age groups (60–64 and 65–75 in Afsin-Elbistan, 55–59, 60–64 and 65–75 in Kutahya-Tavsanli, and 55–59 and 60–64 in Ankara) are confirmed to have statistically significantly lower valuation for risk reduction than their base age-groups (30–34 for Afsin-Elbistan and Kutahya-Tavsanli, and 35–39 for Ankara). In other words, we have confirmed the differences in risk preference between 54 and younger and older age groups. 

Furthermore, the calculated VSL values based on Model 7 are revealed to be negative for age groups 55–59, 60–64 and 65–75 in Kutahya and 55–59 and 60–64 in Ankara. Negative WTP could be interpreted as 1 in 10,000 risk reduction policy is “bad”, not “good” for this age group. One of the possible reasons for this low or negative WTP value could stem from the existing “status-quo bias” among seniors [39,40].

In order to test the status-quo bias among seniors, we included three status-quo cross terms with elderly age dummies (55–59, 60–64 and 65–75) in Model 8 in addition to the variables in Model 7. This model is intended to separate the status-quo preference from risk evaluation for senior age groups. The result of the model reveals the disappearance of, or weakened senior discount effect after separating the preference for the status-quo option for the Kutahya-Tavsanli and Ankara case, but not for the Afsin-Elbistan case. The possible causes of “senior discount” could vary, but the stronger preference for the status-quo option among elderly seems to be one of the reasons. As for Kutahya-Tavsanli, no RISK cross terms are statistically significant and AGE6575 × ASC_SQ is positive significant at the one percent level. As for Ankara, AGE6064 × ASC_SQ and AGE6575 × ASC_SQ are statistically significant at the 1% and 10% levels, respectively, indicating the status-quo preference. The signs of AGE5559 × ASC_SQ are all negative and AGE6064 × ASC_SQ and AGE6575 × ASC_SQ are all positive for all locations. This result shows clear changes in the status-quo preference around age sixty. When we separate the status-quo preference from risk valuation, the “senior discount” disappeared (statistically insignificant risk cross terms for AGE5559, AGE6064 and AGE6575 from the Kutahya-Tavsanli and AGE6064 from the Ankara cases). As a result, the corresponding VSL for the relevant age groups became higher. According to Table 12, the VSL values for all age groups are statistically indifferent from VSL for their base age group (30–34 for Kutahya-Tavsanli and 35–39 for Ankara), except for the 55–59 age group in Ankara. 

**Table 11:** Estimated Result of Age Models—Model 7 and Model 8.

	Model 7						Model 8					
Mean Parameters	Afsin-Elbistan		Kutahya-Tavsanli		Ankara		Afsin-Elbistan		Kutahya-Tavsanli		Ankara	
RISK	1.029	***	1.521	**	0.842	***	1.057	***	1.494	**	0.703	**
	(0.294)		(0.626)		(0.315)		(0.312)		(0.715)		(0.299)	
DATE	−2.926	***	−5.973	***	−2.950	***	−3.039	***	−7.260	***	−2.767	***
	(0.591)		(1.226)		(0.696)		(0.647)		(1.765)		(0.659)	
LUNG	2.687	***	4.838	***	4.286	***	2.806	***	5.799	***	4.074	***
	(0.599)		(1.154)		(1.101)		(0.659)		(1.715)		(1.037)	
CANCER	1.243	***	1.316		2.666	***	1.287	**	1.881		2.474	***
	(0.467)		(0.860)		(0.768)		(0.495)		(1.190)		(0.721)	
TRAFFIC	−4.564	***	−11.980	***	−2.446	***	−4.762	***	−13.600	***	−2.315	***
	(1.160)		(2.828)		(0.874)		(1.258)		(3.529)		(0.815)	
*Fixed Parameters*												
ASC_SQ	−6.015	***	−9.567	***	−5.492	***	−6.229	***	−11.600	***	−5.802	***
	(1.072)		(1.987)		(1.232)		(1.166)		(2.903)		(1.273)	
PRICE	−0.007	***	−0.011	***	−0.006	***	−0.007	***	−0.010	***	−0.006	***
	(0.001)		(0.003)		(0.002)		(0.001)		(0.004)		(0.002)	
Cross Terms with ASC_SQ																					
AGE5559							−1.003		−2.610		−0.616	
							(1.631)		(2.199)		(1.166)	
AGE6064							0.950		0.363		7.225	***
							(0.871)		(2.510)		(2.277)	
AGE6575							0.181		8.500	***	2.508	**
							(1.100)		(2.824)		(1.186)	
Cross Terms with RISK																					
AGE1824	−0.513		−0.311		−0.436		−0.531		−0.080		−0.405	
	(0.363)		(0.773)		(0.444)		(0.375)		(1.106)		(0.422)	
AGE2529	−0.504	*	−1.155		−0.042		−0.518		−0.650		−0.028	
	(0.291)		(0.747)		(0.371)		(0.301)		(1.019)		(0.348)	
AGE3034					−0.451						−0.418	
					(0.346)						(0.337)	
AGE3539	−0.578	*	−0.672				−0.599	*	−0.730			
	(0.321)		(0.763)				(0.335)		(0.883)			
AGE4044	−0.489		−0.371		−0.135		−0.499		0.341		−0.103	
	(0.317)		(0.729)		(0.351)		(0.326)		(0.864)		(0.334)	
AGE4549	−0.666	**	−0.568		−0.188		−0.688	**	−0.740		−0.158	
	(0.320)		(0.798)		(0.342)		(0.335)		(0.963)		(0.328)	
AGE5054	−0.657	*	−0.831		−0.206		−0.681	*	−0.700		−0.173	
	(0.376)		(0.764)		(0.333)		(0.402)		(0.922)		(0.327)	
AGE5559	−0.374		−1.524	*	−1.157	**	−0.541		−1.970		−1.218	**
	(0.437)		(0.885)		(0.511)		(0.513)		(1.239)		(0.531)	
AGE6064	−0.642	*	−1.700	**	−1.665	***	−0.543		−1.170		−0.097	
	(0.350)		(0.855)		(0.604)		(0.370)		(1.327)		(0.529)	
AGE6575	−0.971	**	−3.187	***	−0.537		−0.975	*	−1.700		−0.089	
	(0.449)		(0.836)		(0.450)		(0.507)		(1.261)		(0.482)	
Log L	−991		−946		−610		−990		−935		−596	
PseudoR^2^	0.3		0.34		0.35		0.3		0.35		0.36	
N. of Obs.	1283		1305		850		1283		1305		850	

***, **, and * stand for the statistical significance at 1%, 5% and 10% levels, respectively. The values inside parentheses are standard errors. The estimated results of standard deviation parameters are omitted from the table due to the space constraint. All the estimates are statistically significant at one percent level.

The VSL for the 55–59 age group is calculated based on the coefficient statistically significant at five percent level as −895,647. We have checked for outliers and outstanding characteristics of each observation in this age group, but did not find anything abnormal. Different model specifications have also been tried, but statistically significant AGE5559 × RISK terms were always found. This unexpected result may stem from the fact that the percentage of respondents who are not working, including housewives and retired, increases significantly from 49% for 50–54 to 84% for the 55–59 age group, and as a result, the average household income decreases from 2932 TL to 2232 TL between these two age groups. Since the retirement age is 60 in Turkey, a remarkable increase in the “not-working” proportion of the 55–59 age group is due to the individuals who decided to retire early. We imagine that this age group is in a transition phase from being labor force, to being not-in-labor-force and people may be under the influence of a “significant income loss” and in a psychological stage of “adjusting the reference point” [41]. Observing the fact that the average household income for 60–64 and 65–75 are even lower (2156 TL, 2076 TL) but with no unexpected pattern, it is possible that there is psychological difference between 60 and above (retired at normal retirement age) and 55–59 (could have worked some more years, but decided to retire early, therefore experienced “income loss” by own decision). Although it is not statistically significant, we observe negative VSL for the 55–59 age group for Kutahya-Tavsanli. However, the difference between the average household income before 55 and 55–59 is not as notable for Kutahya-Tavsanli (around 250 TL) as for Ankara (more than 600 TL). As for Afsin-Elbistan, because of the small difference between the average household income before 55 and 55–59 (approximately 300 TL) and the high background health risk condition, we do not observe the same pattern. 

As for Afsin-Elbistan, none of the status-quo cross terms have sufficient explanatory power for this case. This fact could be related to the strong desire among the residents for the policy change regarding air pollution in the area. the VSL for 30–34 age group is 1,592,593 TL while it decreases to 556,424 TL for the 45–49 age group and 123,498 TL for the 65–75 age group. The VSL for the 65–75 age group is approximately one tenth of the peak VSL at the 30–34 age group. This confirms the existence of “senior discount” in this case. However, the amount of discount became smaller once the status-quo bias was removed.

**Table 12:** Estimated VSL from Model 8 (in TL).

Age Groups	Afsin-Elbistan	Kutahya-Tavsanli	Ankara
18–24	792,120	1,059,927	518.806
25–29	812,626	634,545	1,173,728
30–34	1,592,593 *	1,122,312 *	883,532
35–39	690,042	577,158	946,290 *
40–44	840,442	1,378,791	1,042,774
45–49	556,424	568,619	947,753
50–54	566,088	597,174	921,355
55–59	777,936	−358,210	−895,647
60–64	774,095	243,716	1,054,191
65–75	123,498	−151,083	1,068,427

* These values are the base (= calculated for the omitted age categorical dummy variable, 30–34 for Afsin-Elbistan and Kutahya-Tavsanli, 35–39 for Ankara). Bold values are based on statistically significant coefficients theoretically, non-bold values are the same as the base VSL.

## 6. An Application to Air Pollution Policy Evaluation

Since the main objective of this study is to estimate the monetary value of the benefit of an air pollution reduction policy in Turkey, we now apply the estimated VSLs to the specific policy setting, focusing on the new standard for PM_10_. The premature mortality per 10 µg/m^3^ of PM_10_ is estimated by Ostro [42] as 6.72 per 100,000. The three year average (2009–2011) of PM_10_ levels are 100, 84 and 63 μg/m^3^ for Afsin-Elbistan, Kutahya-Tavsanli and Ankara, respectively. Therefore, the expected reduction in PM_10_ levels by 2019 are 60, 43 and 24 in Afsin-Elbistan, Kutahya-Tavsanli and Ankara, respectively. For these levels of reduction, the reduced premature deaths per 100,000 using the coefficient estimated by Ostro [42] are calculated as 6.72 × (60/10) = 40.32 per 100,000 person in Afsin-Elbistan. Since the total population of Afsin-Elbistan is 220,985 according to the 2000 census, the total actual number of premature deaths avoided is calculated as 40.32 × 2.2 = 88.704. Since the VSL calculated from Model 1 is 854,420 TL for Afsin-Elbistan, the welfare gain of the new air quality standard in terms of PM10 is derived as 88.704 × 854,420 = 76,000,574 TL (50,202,601 PPP adjusted USD). For Kutahya-Tavsanli and Ankara, the welfare gains are 47 million TL (31 million PPP adjusted USD) and 441 million TL (292 million PPP$), respectively. The summary of these calculations is given in Table 13.

If we use the EU recommended VSL value of €1.4 million (in 2000 Euros) or $1.72 million (in 2012 USD) in this evaluation, the welfare gains from PM10 reduction are overstated with $182 million (in PPP adjusted 2012 USD) for Afsin-Elbistan and Kutahya-Tavsanli and $1.3 billion for Ankara, that is 3.6 to 5.9 times more than the values our research suggests. Although Turkey is one of the candidate countries of EU, it is revealed that a direct transfer of parameters used for cost-benefit analysis or policy evaluations in EU countries could lead to misleading conclusions. In addition, benefits transfers based on studies conducted in North America or Western Europe with a simple income-based adjustment to the sites in developing countries have a high potential to derive unreliable estimate of VSL since the existing health risk conditions might have stronger influences on WTP and VSL than the differences in household income between the two locations, as we observed in the Afsin-Elbistan and Kutahya-Tavsanli cases, and existing health and environmental risks are typically higher in developing countries.

**Table 13:** Estimated VSL Application to Air Pollution Policy Evaluation.

Components of Welfare Gain Derivation	Afsin-Elbistan	Kutahya-Tavsanli	Ankara
2009–2011 Average of PM_10_ (μg/m^3^)	100	83	64
Target Reduction by 2019 (40 μg/m^3^)	60	43	24
Reduced Premature Mortality per 100,000 (6.72 per 10 μg/m^3^)	40	29	16
Population (2000)	220,985	307,680	4,007,860
Reduced Number of Premature Death	89	89	641
VSL (TL)	854,420	527,878	689,104
Welfare Gain from PM_10_ Reduction (TL)	76,000,574	47,144,387	441,827,549
Welfare Gain from PM_10_ Reduction (PPP, USD)	50,202,601	31,141,487	291,851,642

## 7. Discussion and Conclusion

We have estimated the value of statistical life (VSL) for Afsin-Elbistan, Kutahya-Tavsanli, Ankara, and the pooled case in this study, and obtained 0.85, 0.53, and 0.69 million TL or 0.56, 0.35, 0.46 and 0.49 million PPP adjusted 2012 dollars based on the base models. The WTPs for 1 in 10,000 reduction of the mortality risk are estimated as 85 TL ($56), 53 TL ($35), and 69 TL ($46) for Afsin-Elbistan, Kutahya-Tavsanli, and Ankara, respectively. We also confirmed that the VSLs based on different risk types could result in type-specialized VSL values that are different from the general VSL calculated from an arbitrary cause of risk reduction. Different types of cancer also cause different VSL estimates. The lung cancer premium as the averaged result from three regions is found to be 213% against traffic accident. The effects of a one-year-delayed provision of the risk-reduction service are the reduction of WTP by 482 TL ($318) per person on average, and the disutility from the status-quo (zero risk reduction) against alternative is found to be 891 TL ($589) per person on average. We found no evidence of gender effect on risk or status-quo option evaluation. The households with higher income prefer risk reduction options against the status-quo (Afsin-Elbistan, Ankara), and are willing to pay more for the risk reduction (Kutahya-Tavsanli). For an increase in 100 TL in the monthly household income, the WTP for risk reduction increases by 6.3 TL in Kutahya. University graduates are willing to pay around 100 TL more for 1 in 10,000 risk reduction while the good health condition tends to make people value risk reduction about 55 TL ($36) less. We did not find any evidence of the influence of existing respiratory illnesses on the individual preference over risk reduction while the diseases cause an increase in the willingness to pay of 644 TL ($425) more on average for the risk reduction option. 

As for the controversial senior discount, we confirmed that certain senior age groups value risk reduction statistically significantly less than the younger age groups. However, we confirmed that such lower valuation of the risk reduction is partly due to the strong preference toward the status-quo option among elderly. The inclusion of status-quo cross terms with three senior age groups led to the disappearance of the “senior discount” for the Kutahya-Tavsanli and Ankara cases and decreased the magnitude of the senior discount for the Afsin-Elbistan case. In fact, we found no age-effect on risk evaluation for Kutahya-Tavsanli and Ankara (except for the 55–59 age group). Therefore, it is observed that the status-quo bias among elderly people is a partial cause of the lower estimated WTP for risk reduction and lower VSL. We also learned from the Afsin-Elbistan case that the existing environmental health risk situation strongly affects the risk evaluation and the preferences for risk reduction policies. Contrarily, the VSL values for the peak age-groups (30–34 for Afsin-Elbistan and Kutahya-Tavsanli and 35–39 for Ankara) are 1.6 million TL (1 million PPP adjusted USD), 1.1 million TL (0.7 million PPP adjusted USD) and 1 million TL (0.7 million PPP adjusted USD) for Afsin-Elbistan, Kutahya-Tavsanli and Ankara, respectively, or on average, 1.25 million TL (0.8 million PPP adjusted USD). 

As the result of the reduction of PM_10_ to the EU air quality standard level of 40 µg/m^3^, the premature death reduces by 89 cases in Afsin-Elbistan and Kutahya-Tavsanli and by 641 cases in Ankara. The total estimated welfare gain from the policy in these study areas in terms of reduced premature death is 565 million TL or 373 million PPP-adjusted dollars.

Lastly, the feasibility of conducting a choice experiment in Turkey is confirmed if it is conducted as an in-person interview. Considering the relatively high illiteracy rate especially among females and the relatively low level of education especially in smaller cities, mail or internet surveys are not reasonable options at this point in Turkey. However, if it is conducted in person, considering the fact that 80% to 90% of the respondents considered “understanding hypothetical question” as easy or very easy, and 90% to 95% of the respondents expressed their confidence over their answers to the hypothetical question, it is possible to obtain reliable responses even in a small city such as Afsin.

## Figures and Tables

**Figure A1:**
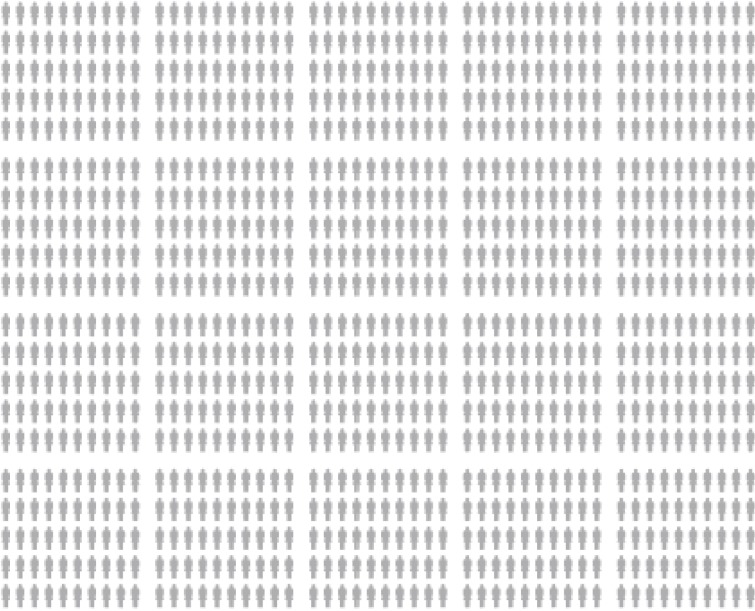
An Example of 10,000 Human-Shaped-Grid (Partial) for Risk Communication.

## Appendix

### A: Choice Experiment Question

Currently in urban cities in Turkey, about 20,000 people die from respiratory related diseases and 100,000 people die from heart related diseases every year. Every year, about 70,000 people diagnosed as cancer, and 30% of the cancer incidences are lung cancer. Reducing the risk of early death costs time and money. In order to reduce the risk of early death due to cancer, for example, careful daily diet and regular exercises together with regular screenings and medical check-ups are necessary. It is also costly to reduce the probability of experiencing traffic accidents. The cost includes careful driving, wearing seatbelts, purchasing/installation of air bags, and regular maintenance.

In this question, we would like to ask you which options you would prefer if there is a chance to reduce the probability of early death with specific causes such as respiratory diseases, lung cancer, other type of cancer, and traffic accident. You will be shown two options with different cause of death, if the risk reduction is about yourself or about one of your children, if the reduction is in effect immediately or after 1 year, risk reduction amount for one year and the payment to realize the risk reduction. Please compare Option A and Option B and decide which one you will prefer and actually willing to participate in such a program by paying the specified amount (Table A1).

**Table A1:** An example of a choice set.

Attributes	A	B
Risk Type	Respiratory Disease	Lung Cancer
Affected Person	Own	Own
Effective Date	Today	1 year later
Risk Reduction (for 1 year)	8/10,000	5/10,000
Price (for 1 year)	800 TL	600 TL

Which one would you choose? A or B, or neither? (Answer: )

### B: 10,000 Human-Shaped-Grid

In the survey, interviewers displayed a separate A4 sized paper with 10,000 human shapes (Figure A1) to each respondent and explained how 1/10,000, 3/10,000, 5/10,000 and 8/10,000 look like for the clearer risk communication.
